# In-situ porosity recognition for laser additive manufacturing of 7075-Al alloy using plasma emission spectroscopy

**DOI:** 10.1038/s41598-020-75131-4

**Published:** 2020-11-10

**Authors:** Wenjing Ren, Jyoti Mazumder

**Affiliations:** 1 Department of Mechanical Engineering, Xi’an Jiaotong University, Xi’an, 710049 Shaanxi China; 2 Department of Mechanical Engineering, University of Michigan, Ann Arbor, MI 48109 USA

**Keywords:** Mechanical engineering, Laser material processing

## Abstract

Poor quality and low repeatability of additively manufactured parts are key technological obstacles for the widespread adoption of additive manufacturing (AM). In-situ monitoring and control of the AM process is vital to overcome this problem. This paper describes the combined artificial intelligence and plasma emission spectroscopy to identify the porosity of AM parts during the process. The time- and position-synchronized spectra were collected during the directed energy deposition (DED) manufacturing process of a 7075-Al alloy part. Eighteen features extracted from spectra were coupled with the deposition qualities which were characterized by the 3D X-ray Computed Tomography (CT) scan and used to train a Random Forest (RF) classifier. The well-trained RF classifier achieved up to 83% precision for the porosity recognition of depositions. The feature importance recorded by the RF classifier indicates that the intensities of spectra at the wavelength of 414.234 (Fe I) nm and 396.054 (Al I) nm, and the kurtosis of spectra at wavelength ranges of 484–490 nm and 508–518 nm, are the most effective features for porosity recognition. The physical correlations between spectra, porosity formation, and thermal accumulation during the AM process were analyzed. This study demonstrates the great potentials, as well as challenges of plasma emission spectroscopy for in-situ quality monitoring of laser AM which allows the enhancement of AM technique.

## Introduction

Additive manufacturing (AM), hailed as one of the most innovative technologies in Industry 4.0, is leading to innovations in a wide range of sectors, including aerospace, automotive, and medical^1,2^. However, the low repeatability of qualified parts prevents AM from being fully adopted in practical applications, especially in industries with strict requirements on parts quality^3,4^. Traditional post-build part characterization methods, like X-ray computed tomography (CT), are time- and cost-consuming and their implementations are limited by the scale of production^5^. Defect detection during the AM process using in-situ monitoring techniques is an essential way to overcome these challenges. Signal analysis of the laser-metal interaction zone in real-time allows for defects and mechanical properties to be detected and corrected in real-time without interruption^6,7^. In-situ monitoring is urgently demanded by AM since conventional statistical quality control methods are not always applicable for the quality assurance of AM due to its low volume production and high variants of products.

This study focus on the in-situ recognition of internal porosity in laser additive manufactured parts. Internal porosity has fatal effects on mechanical properties and reliability and is difficult to detect in-situ due to the complex formation mechanisms below the build surface^8^. Although some popular optical sensors including pyrometers and cameras have been shown to be effective for detecting geometric defects such as deformation and unqualified surfaces^9,10^, they cannot provide much fundamental physics information except the temperature and images of the build surface, and thus are unreliable for internal defect detection. Coeck et al.^11^ employed the light intensity collected during the powder bed fusion (PBF) process using photodiodes to monitor non-fusion type porosities induced by the suboptimal gas flow. An anomaly of the light intensity was observed in the location where the CT scan showed a large pore. Similar to Coeck’s work, the present investigations of in-situ porosity recognition mainly focus on the non-fusion type porosity caused by abnormal process parameters. The principle in these studies is that non-fusion type porosity mainly occurs along with changes of geometry or temperature in the melt pool which can be detected by optical cameras or thermal sensors^12^. However, porosity formation mechanisms are various and complex^8^. Pores commonly occur in AM parts even under optimal parameters through either entrapped gas or hydrogen dissolution due to the thermal density variation or anomaly in the manufacturing process, especially for the 7075-Al alloy which has high thermal conductivity and complex compositions including volatile materials such as Mg and Zn. For these types of porosity, signals which provide more fundamental information related to the porosity are needed. Recently, Leung^13^ demonstrated the application of high-speed synchrotron X-ray imaging for porosity monitoring in laser PBF. This research revealed the pores formation mechanism and pores’ dynamic behaviors under the build surface. However, the X-ray imaging technique can only be used for small samples for limits of X-ray image resolution, which hinders its adoption in practical applications together with the high cost of synchrotron X-ray implementation.

To overcome the above challenges in in-situ porosity detection, an advanced sensing technique named emission spectroscopy is employed in this study. The emission spectra are made up of photons emitted from excited atoms in the plasma generated during the laser AM process. Recorded with fine wavelength resolution, emission spectra contain much more physical information than other optical signals. The spectral emission wavelength reveals the chemistry of the plasma and the spectral intensity reflects the element concentrations and plasma conditions such as plasma temperature and electron density^14^. Owing to the rich information in the spectra, emission spectroscopy has the potential for monitoring multiple part qualities, from defects to composition. Research using emission spectroscopy on in-situ monitoring of laser AM has been done before, notably by Mazumder and his group members who have been carrying out an in-depth study on this topic for a considerable amount of time. They have demonstrated that emission spectroscopy is effective for in-situ monitoring of feedstock composition^15^, phase transformation^16^, and residual stress^17^ of parts. In their recent patents, spectral features including spectral line intensity, line-to-line ratio, and plasma temperature are used for the closed-loop control in a Direct Energy Deposition (DED) system^18^. Lough^19^ also equipped a spectrometer into the PBF system to measure the real-time spectra for melt pool size monitoring. Stutzman et al^20^ demonstrated that the median line-to-continuum ratios of spectra at 430 nm and 520 nm significantly increased when the DED part presented non-fusion pores induced by suboptimal process parameters. However, this study doesn’t detect the porosity quantitively and the physical relationship between pores and spectra were not analyzed. Montazeri et al^21^ developed a machine learning model for porosity detection of laser PBF using the line-to-continuum ratio of Cr spectral emission. The proposed method can accurately recognize the porosity level of parts printed under different process parameters which has proved the great potential of spectroscopy for porosity recognition. The main limitation of this study is that it recognizes the porosity layer-by-layer which makes it not applicable for real-time defects correction.

A challenge in using this method is knowing which features in the spectra give the most meaningful information about the part quality. Various features can be extracted to interpret spectra in a different way, such as emission intensity, intensity ratios, and spectral profile properties. However, the importance of these spectral features for porosity detection has not been discussed. The physical principle of emission spectroscopy on defects recognition needs to be studied further. Therefore, an artificial intelligence method named Random Forest (RF) classifier has been employed in this study, which can estimate the importance of individual spectral features during developing the porosity recognition model. In summary, this study investigated the in-situ recognition of local porosity in the laser-based DED part produced under consistent parameters. Spectra signals were related to the porosity defects which were characterized by 3D X-ray Computed Tomography (CT). An intelligent RF classifier was developed for local porosity recognition using spectral features. Important spectral features for porosity detection were determined and analyzed.

## Methodology

### Plasma emission spectroscopy in laser AM

**Figure 1:**
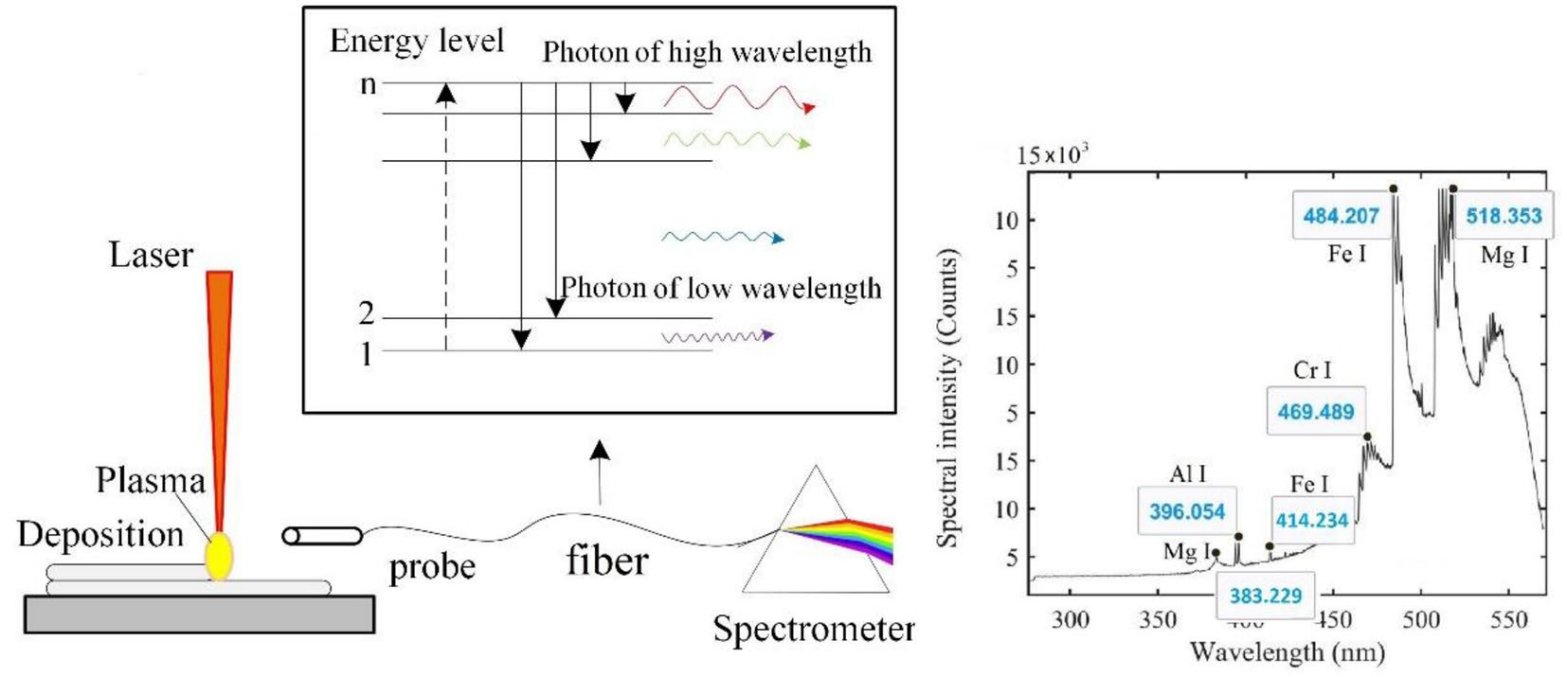
Plasma emission spectroscopy in laser AM.

Unlike the laser-induced breakdown spectroscopy (LIBS), spectra are directly produced by the laser in the additive manufacturing process. As shown in Fig. 1, metal deposition and powder are melted and partially evaporated under the illumination of a highly energetic laser. Atoms in the metal vapor and the shielding gas are excited to high energy level states and then transit to lower energy level states based on spontaneous transition theory^22^. During these spontaneous transitions, photons with specific wavelengths determined by the energy gaps of transitions are released and recorded as emission spectra. Since the energy gap is a characteristic of each element, the wavelengths of the emission spectrum are identifiers for the radiating atoms. Further information on emission spectroscopy can be found in references^14^. The spectral plot in Fig. 1, for instance, is a spectrum collected during laser additive manufacturing of 7075-Al alloy. Peaks at wavelengths 383.229 nm, 396.054 nm, 414.234 nm are respectively identified as the peaks of Mg I, Al I, and Fe I which are the main elements in the target material.

The intensity of the spectrum is proportional to the density of emitted photons. Under the local thermal equilibrium assumption, the emission density (${I}_{{i}_{j}}(\lambda )$) of photons is:

1 $$\begin{array}{c}{I}_{{i}_{j}}\left(\lambda \right)=\frac{1}{4\pi }{n}_{0}{A}_{ij}\frac{{g}_{i}{\mathrm{e}}^{-{E}_{i}/{k}_{B}T}}{U\left(T\right)}I\left(\lambda \right) \end{array}$$

where the partition function $U\left(T\right)$ is the statistical occupation fraction of every level of the atomic species:

2 $$\begin{array}{c}U\left(T\right)=\sum_{j}{g}_{j}{e}^{-{E}_{j}/{k}_{B}T}\end{array}$$

There are two types of variables: (1) the element-determined variables, including the wavelength of the photon ($\lambda$), the transition probability (${A}_{ij}$), the degeneracy of the upper level (${g}_{i}$), the energy at level $i$ (${E}_{i}$) and level $j$ (${E}_{j}$); (2) and the plasma-determined variables, including the number of neutral atoms in plasma (${n}_{0}$), the temperature of plasma ($T$), and the spectral line profile ($I(\lambda )$). These variables are directly correlated with process parameters and the quality of the manufactured parts. For example, the laser power density affects the temperature and electron density of the plasma, which in turn affects the intensity and profile of spectra. Parameters, including laser properties (wavelength and power distribution), powder delivery rate, and shielding gas flow also influence the spectral properties significantly. Therefore, the close relationships between spectra signal and manufacturing parameters and quality make emission spectroscopy a great candidate for in-situ defects detection.

### Theory of random forest

**Figure 2:**
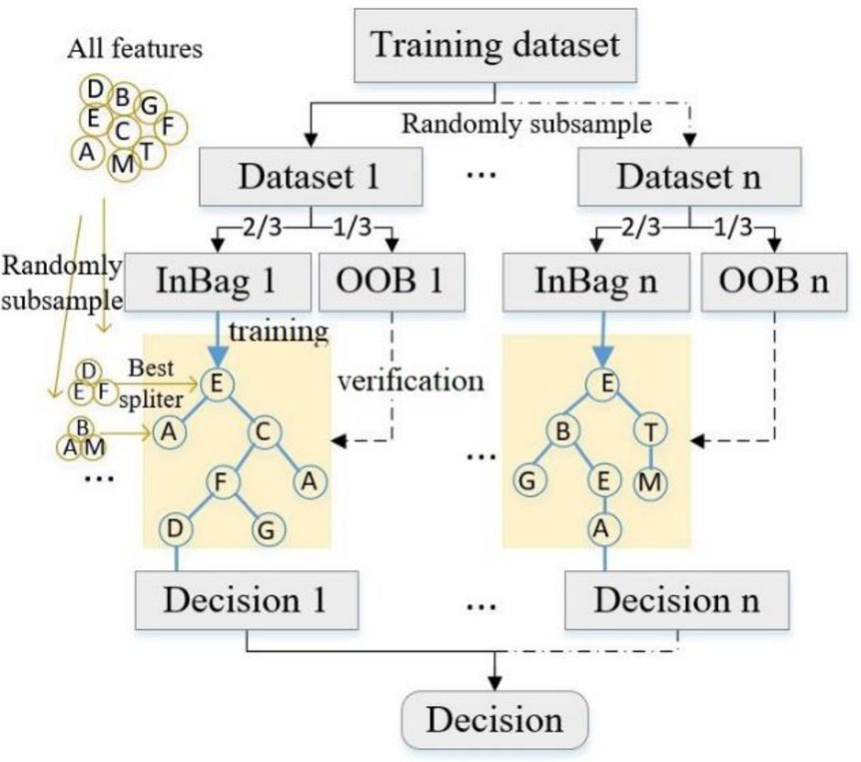
Random Forest schematic diagram.

Random Forest (RF) is an ensemble model that is constructed by multiple classifications or regression trees (CARTs) and makes predictions based on majority voting from individual trees^23^. The building procedures of an RF classifier are shown in Fig. 2 in which a two-stage randomization procedure contributes unique advantages of the RF model. One is that examples used to grow individual decision trees are randomly selected from the training dataset with replacement, known as bootstrap aggregating^24^. By assembling trees grown from different training data subsets, the RF model becomes more robust when facing slight variations in input data and, in turn, achieve greater classification stability. Several studies have proved that models based on bootstrapping, like RF, are less sensitive to noise and more effective for handling imbalanced data compared to other models^25,26^. Also, as growing trees, only 2/3 of the bootstrapped dataset is used to train the current tree and the other 1/3 of the dataset forms another subset named out-of-bag (OOB). The OOB dataset is used to get an unbiased estimate of the classification error as trees are added, which avoids the RF model overfitting data. The OOB method has a similar function with cross-verification but almost no additional computational task is needed. Another randomization stage is that rather than using all features only a random subset of features is used as candidates for splitting each node of a tree. This can reduce the correlation between trees so that RF gets a higher generation accuracy although the strength of an individual tree is decreased.

As growing trees, a feature selection measurement is required to split examples at each node. One of the most popular measurements, named the Gini impurity criterion, is used in this study. The Gini impurity indicates how pure the node is, and it goes to zero when all examples at the node are purely classified. At each node, the decision tree searches through the subset features for the value to split on that results in the greatest reduction in Gini impurity. The Gini impurity at node *m* ( ${GI}_{m}$) of all classes is calculated using Eq. (3), where, ${f}_{i}$ is the probability of class $i$ at *m* node and C is the number of the unique classes.

3 $$\begin{array}{c}{GI}_{m}={\sum }_{i=1}^{\mathrm{C}}{f}_{i}\left(1-{f}_{i}\right)\end{array}$$

RF also provides an assessment of the relative importance of each feature for classification. This property is significant because it is important to know how each feature influences porosity recognition, and which feature is most relevant to porosity defects. To get the relative importance of each feature, the RF model replaces one of the input random features and measures the resulting decrease in accuracy by seeing the change in the OOB error estimation and the Gini impurity^24^. Investigating the response of the most important features to additive manufacturing quality is helpful to reveal the underlying fundamentals of defects formation.

## Experiment

**Figure 3:**
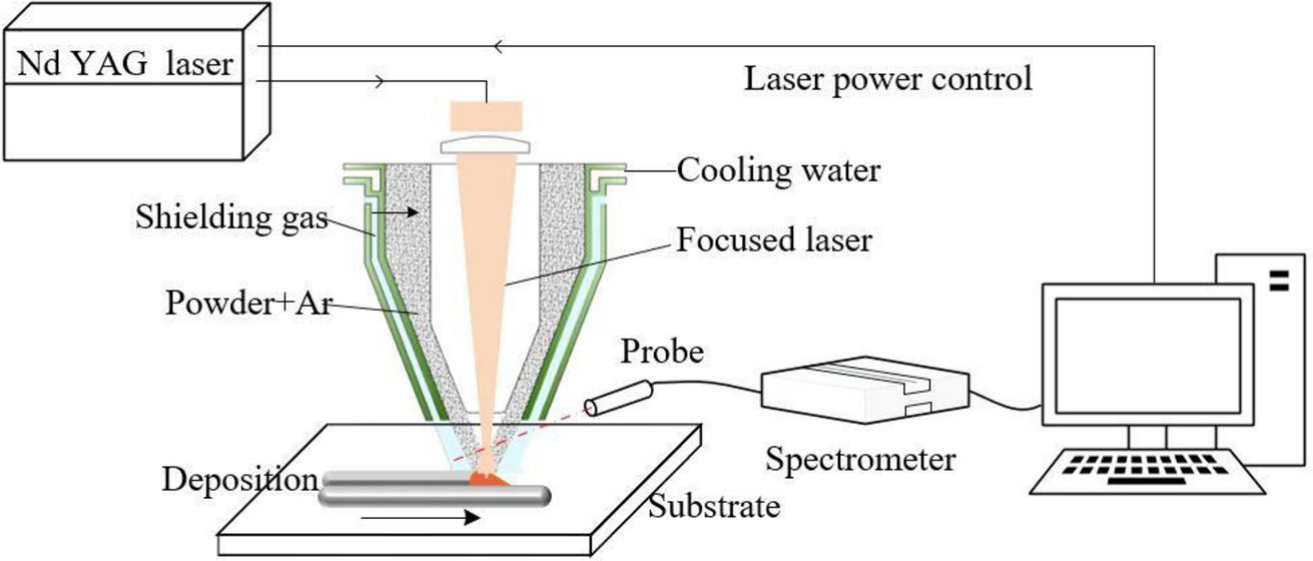
Schematic diagram of the DED system with SOMS.

Experiments were conducted using the smart DED system developed at SenSigma company, USA. The DED system consists of a high power Trumpf 6 kW Nd: YAG laser, a six-axis robot, a material supplier, and a Smart Optical Monitoring System (SOMS). The experimental setup is shown in Fig. 3. A Gaussian laser beam with a wavelength of 1030 nm is focused on the build surface. An optical head and a customized nozzle are assembled on the robot arm. Argon gas is used to shield the powder flow into the laser beam, and create an inert environment around the metallic deposition. The 7075 powders are delivered co-axially with the laser beam and are melted and deposited as the delivery nozzle moving relative to the base plate. An optical probe collects the light emitted from the plasma and transmits the light to the spectrometer (Ocean Hr2000). The optical probe is set to be nearly parallel to and 2 mm above the substrate, and 150 mm away from the laser beam. Spectra are collected during the DED process using the SOMS system with a spectrometer having a 277–570 nm wavelength range and a resolution of 0.154 nm. The integration time of the spectrometer is set to be 0.01 s.

**Figure 4:**
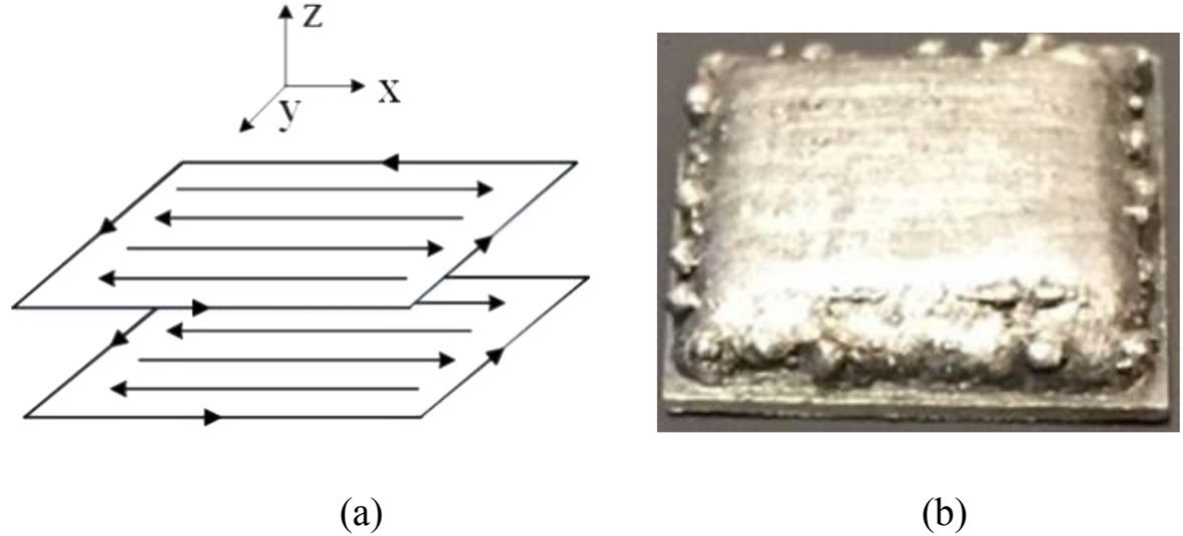
Printing path **(a)** and the as-deposited specimen **(b)**.

The 7075 powder is gas-atomized with a particle size ranging between 44 $\upmu$m and 105 $\upmu$m (− 140/ + 325 mesh). Table 1 presents the chemical composition of the powder. To print a part with qualified dimensions and containing the proper number of pores, optimization experiments were conducted following the Taguchi design method with laser power, scanning speed and the powder delivery rate between 1000–1200 w, 6–8 mm/s, and 0.5–1.5 g/min, respectively. Only the parameters as listed in Table 2 were used to print the part for the porosity recognition investigation in this study, as all other parameters produced parts with unacceptably high numbers of pores. Figure 4a schematically illustrates the scanning path, which consists of 10 layers and 14 bidirectional lines in each layer. The layer thickness and hatching space are set to be 0.35 mm and 0.75 mm, respectively. At the beginning of each layer, a square periphery would be printed along the edge of the deposition to avoid collapse. The nozzle goes back to the start point after each layer being done, and rises 0.35 mm, then repeats to print the next layer. In this way, a rectangular part, shown in Fig. 4b, with dimensions of $12.6\times 12.6\times 3.5$ mm, was deposited upon a 7075-Al alloy substrate with a thickness of 12.7 mm (1/2 in.). Spectra signals were synchronously collected for each deposition layer.

**Table 1:** Chemical weight composition of 7075 powder.

Elements	Zn	Mg	Cu	Si	Fe	Mn	Cr	Ti	Al
Percentage (%)	5.1–6.1	2.1–2.9	1.2–2.0	0.4	0.5	0.3	0.18–0.28	0.2	Balance

**Table 2:** Process parameters for printing the part for porosity recognition investigation.

Parameter	Laser power	Scanning speed	Powder flow rate	Laser beam	Layer space	Hatching space
Value	1200 (W)	6 (mm/s)	1.5 (g/min)	1.2 (mm)	0.35 (mm)	0.75 (mm)

The printed part was cut off from the substrate using an abrasive cutting machine and inspected in the as-built condition. Post-inspection was implemented for the presence of pores in the part using a 3D X-ray Computed Tomography system (Zeiss Versa 520) with a voxel resolution of 7.5 µm in each direction (x, y, and z). The part was mounted on a high-precision stage and incrementally rotated by 0.225 degrees for a full 360° rotation. As the sample rotating, X-ray beams were transmitted through the part at a power of 7 w and a voltage of 80 kV getting one projection image at each rotated angle with an exposure time of 3 s. The source filter used during the scanning is LE2 glass filter. 1601 projections were taken in 360° of the part and reconstructed to a complete 3D X-ray CT scan representation of the part. The outputs of the X-ray CT characterization were used to manually label the local deposition as dense or porous.

## Experimental data analysis

### Spectra signal possessing

**Figure 5:**
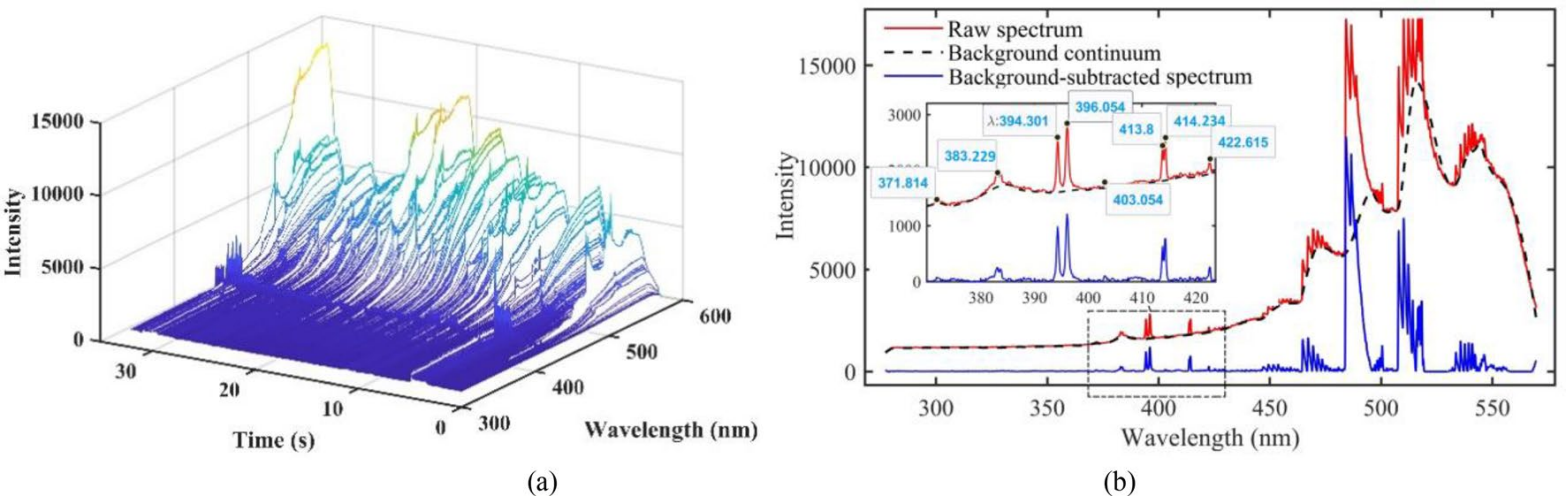
3D plot of an example of spectra collected from one layer **(a)** and spectrum collected over one integration time **(b)**.

The raw spectra signals collected from one layer printing process are plotted in Fig. 5a. There are 3480 spectral frames collected in each layer in which each spectral frame consists of a spectrum with 2047 variables in wavelength dimension. Since the spectra signals are collected synchronously during the printing process, each frame of the spectra can be located at a specific voxel of deposition based on Eq. (4).

4 $$\begin{array}{c}L={N}_{th}\cdot\tau \cdot V\end{array}$$

where *L* is the printing length corresponding to the ${N}_{th}$ frame of spectra. $\tau$ and $V$ are the integration time of spectrometer and printing speed, respectively. Therefore, the spectra data can be labeled with the quality of deposition qualified by X-ray CT scans.

As shown in Fig. 5b, the raw spectrum is made of continuums spectrum (background continuum) and emission spectrum (emission lines) which are produced in completely different mechanisms and hold distinct information about the process. Thus, the background continuum and emission lines were extracted from the raw spectrum and interpreted separately. The first lower envelope of the raw spectrum is calculated using a linear interpolation formula shown in Eq. (5). Then the lower envelope of the first lower envelop curve was calculated in the same way and served as the background continuum of the spectrum.

5 $$\begin{array}{c}P\left(\lambda \right)=f\left(\lambda -\Delta \lambda \right)-\frac{f\left(\lambda +\Delta \lambda \right)-f\left(\lambda -\Delta \lambda \right)}{2}\end{array}$$

where $P\left(\lambda \right)$ is the interpolation at wavelength $\lambda$ and $f\left(\lambda \right)$ is the intensity of the curve to be interpolated at the wavelength $\lambda$. Figure 5b presents the estimated background continuum (black dash line) and the emission lines (blue line) for a raw spectrum (red line). As can be seen, sharp peaks present at 383.229 nm, 396.054 nm, 414.234 nm, which are respectively identified as Mg I, Al I, and Fe I based on the NIST Atomic Spectra Database^27^.

### Spectral feature extraction

The full wavelength range spectrum is too intricate to indicate the processing conditions directly. Therefore, effective and reliable features have to be extracted. Here, eighteen spectral features related to emission lines of Mg I (383.229 nm), Al I (396.054 nm), Fe I (414.234 nm, 473.566 nm, and 518.216 nm), Cr I (484.207 nm), and statistical properties in high wavelength ranges were extracted in various ways. Table 3 presents the details of the eighteen spectral features.

**Table 3:** Features introduction and definition.

Feature No.	Features	Formula
1–5	Background-subtracted emission line intensities	$I\left({\lambda }_{i}\right)={\int }_{{\lambda }_{i}-{d}_{i}}^{{\lambda }_{i}+{d}_{i}}\left({I}_{r}\left({\lambda }_{i}\right)-{I}_{b}\left({\lambda }_{i}\right)\right)d\lambda$ (${\lambda }_{i}$ = 383.229, 396.054, 414.234, 473.566, 484.207 nm, ${d}_{i}$=0.882, 1.168, 1.014, 1.123, 1.813)
6	Average raw spectra intensity	$\frac{1}{N}\sum_{\lambda \epsilon \phi }{I}_{r}\left(\lambda \right)$
7	Average background intensity	$\frac{1}{N}\sum_{\lambda \epsilon \phi }{I}_{b}\left(\lambda \right)$
8	Integration of background continuum	$\int \left({I}_{r}\left(\lambda \right)-{I}_{b}\left(\lambda \right)\right)d\lambda , \lambda \epsilon \phi$
9–12	Line-to-continuum ratios	${R}_{i}=I\left({\lambda }_{i}\right)/{I}_{b}\left({\lambda }_{i}\right)$, (${\lambda }_{i}$=414.234, 396.054, 473.566, 484.207 nm)
13–14	Line-to-line intensity ratios	$I\left(396.054\right)/I\left(414.234\right),$ $I\left(484.207\right)/I\left(518.216\right)$
15–16	Root mean squares of raw spectral intensity in sub-wavelength-ranges	${RMS}_{j}=\sqrt{\frac{1}{{n}_{i}}\sum_{{\lambda }_{i}\epsilon {\varphi }_{j}}{{I}_{r}}^{2}\left({\lambda }_{i}\right)}$, *j* = 1, 2
17–18	Kurtosis of raw spectral intensity in sub-wavelength-ranges	${\kappa }_{j}=\frac{\frac{1}{{n}_{i}}{\sum }_{{\lambda }_{i}\epsilon {\varphi }_{j}}{\left({I}_{r}\left({\lambda }_{i}\right)-{\stackrel{-}{I}}_{r}\right)}^{4}}{{\left(\frac{1}{{n}_{i}}{\sum }_{{\lambda }_{i}\epsilon {\varphi }_{j}}{\left({I}_{r}\left({\lambda }_{i}\right)-{\stackrel{-}{I}}_{r}\right)}^{2}\right)}^{2}}-3$, *j* = 1, 2

Where, ${I}_{r}\left(\lambda \right), {I}_{b}\left(\lambda \right)\, \mathrm{and}\, \space I\left(\lambda \right)$ represents the raw spectral intensity, background intensity, and background-subtracted intensity at the wavelength $\lambda .$ The full wavelength range of the spectrometer $\phi =\left[227, 569\right] (\mathrm{nm})$. The sub-wavelength-ranges for RMS and Kurtosis calculation are ${\varphi }_{1}=\left[484 , 490\right]\, \mathrm{and}\,\space {\varphi }_{2}=\left[508 , 518\right].$ The symbol ${n}_{i}$ refers to the points number in the sub-wavelength-range ${\varphi }_{i}$. The symbol ${d}_{i}$ refers to the haft broaden of the emission line at the wavelength ${\lambda }_{i}$.

Since the laser-material interaction area equals the laser beam diameter, which is 1.2 mm in this study, the prediction resolution was set to be 1.2 × 1.2 mm in the XY plane. Within each interaction area, 20 frames of spectra were collected. An average of 20 frames was taken for each feature and served as the input of the classification model.

### Post-built porosity calculation

The actual quality (ground-truth) of each local deposition in the part is labeled by the X-ray CT scanning results. To correlate the synchronized spectral data with the porosity distributions, the CT scans of the part were manually rotated and translated to register the CT scan volume to the same coordinate system. The CT scanning volume was sectioned into 250 slices perpendicularly to the building direction. Based on the deposition layer thickness, 24 CT scanning slices were assigned to each printing layers and ten slices were assigned to the attached substrate for porosity rate calculations.

**Figure 6:**
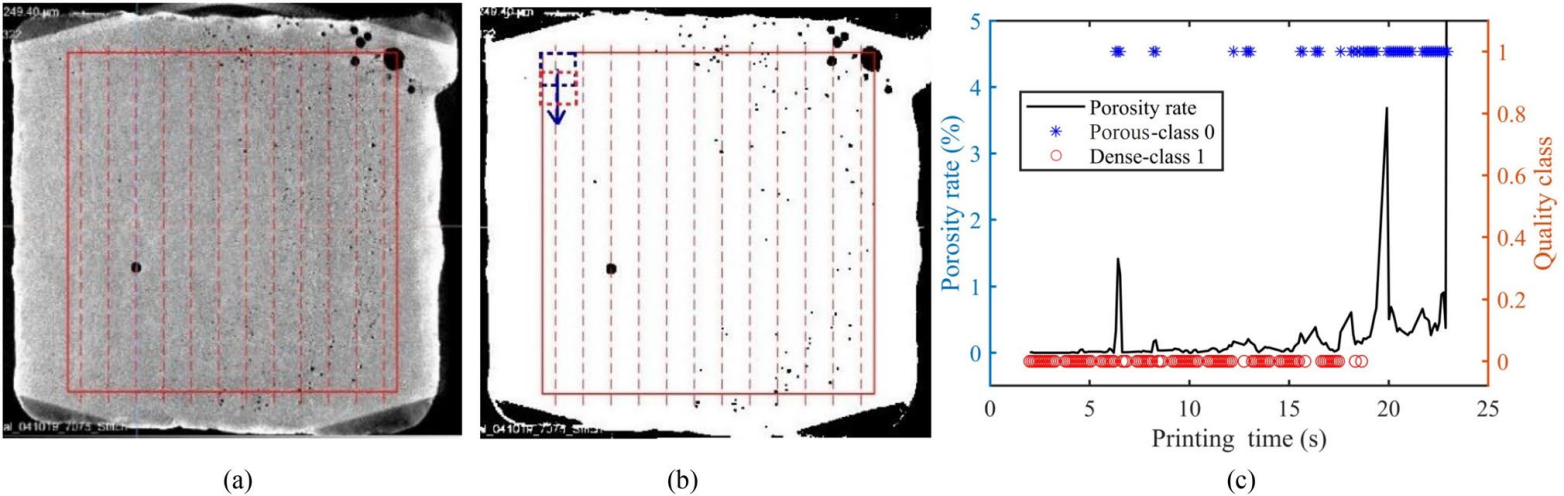
Diagram of the CT scanning image processing procedure: a representative CT image in the second layer **(a)**, the corresponding binary CT scan image **(b)**, and the calculated porosity rate **(c)**.

To eliminate the complications arising from the effects of the edge depositions, analysis of the CT image is limited to a region of interest (AOI) which is approximately 1.8 mm away from the edges. Figure 6a gives a representative slice of CT scans, in which the red box and dash lines illustrate the AOI and the 12 printing paths (from the second line to the thirteenth line), respectively.

The porosity distribution in each layer of the part can be calculated as follows: (1) Convert each slice in one deposition layer to a binary image using a threshold of 140 lightness, so that the pores in the slice are converted into black pixel contrasting to the white dense deposition areas. (2) Get the spatial porosity rates by calculating the ratios of the black pixels to the white pixels in the local area. As shown in Fig. 6b, the local area is outlined by a window with a size of $111\times 111$ pixels, which correspond to an area of $1.2\times 1.2 \mathrm{mm}$ deposition. (3) By moving the window along the printing path with 50 pixels overlap, the spatial porosity rate of the whole slice can be obtained. (4) Calculate the porosity rate of each slice in this layer using the same method. Average the porosity rate at the same position in the XY plane of all slices in this layer. (5) Label the local deposition region as porous (class 0) if the average porosity rate is higher than 0.02%, otherwise, label the deposition region as dense (class 1). Implementing these steps on each layer, the spatial porosity rates of the whole part were calculated, and consequently, each local deposition was labeled. Figure 6c presents the calculated porosity distribution for the second layer, with the horizontal axis representing the printing time.

A combination of spectral features and ground-truth quality of a local region ($1.2\times 1.2 \mathrm{mm}$) is regarded as one example used for the RF classifier training and testing. Consequently, 156 examples are obtained from each layer. To eliminate the effects of the substrate, data from the first layer was discarded. The spectra signals from the eighth layer were not saved due to operator error. Altogether, a dataset consisting of 1092 examples from seven layers (2nd, 3rd, 4th, 5th, 6th, 7th, and 9th layers) was created and used for porosity recognition. Since the part was printed layer by layer, each layer can be taken as an individual part. Therefore, examples from the 2nd layer and the 3rd layer were used to train and test the RF classifier to verify the recognition performance. Examples from the other five layers were used for experimental verification to verify the robustness of the model. Specifically, 75% of the examples in the second and the third layers were used for training the RF classifier. The rest 25% of examples from these two layers and examples from other layers were used for testing. Each testing operation was repeated ten times to ensure the reliability of the results.

## Results and analysis

### Porosity recognition performance

**Figure 7:**
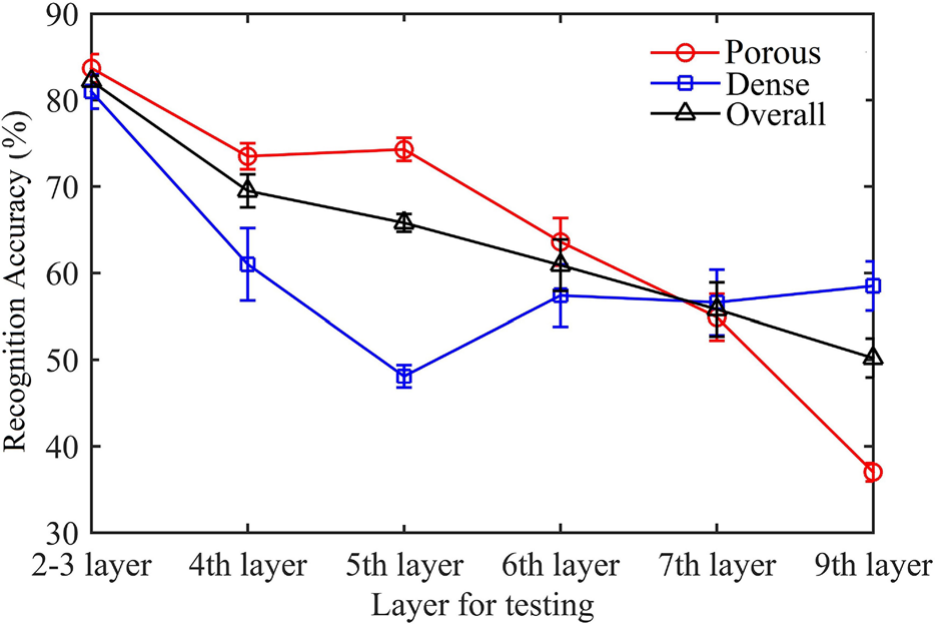
RF testing results for dense and porosity recognition.

Figure 7 compares the recognition performances for porous, dense, and overall examples. The mean values of testing precisions are shown as lines. The precision standard deviations of ten repeat operations are shown as the error bars. Some observations can be found: (1) The classifier trained by the data of the 2nd layer and the 3rd layer can recognize the porosity of other examples of these two layers with relatively high recognition precisions (83% for porous examples, 82% for fully dense examples and 82% for overall examples). The F1-score for the prediction of these two layers is 82%. Table 4 presents the confusion matrix of the test result for these layers. (2) The recognition accuracies for the quality of the subsequent deposition layers decrease layer after layer. Furthermore, the recognition accuracies of the training layers and the layers close to the training layers have shown a lower standard deviation than the layers far behind the training layers. (3) The precision for recognizing the porous examples is higher than the precision for recognizing the dense examples, except for the last two layers.

**Table 4:** Confusion matrix of the test result for the 2nd and 3rd layers.

Example number = 78	Actual porous	Actual dense
Predicted porous	31	6
Predicted dense	7	34

The decrease of the recognition performance for the subsequent layers is mainly caused by the variability in deposition thickness. In this study, the probe of the spectrometer is assembled on the optics head and focused on a specific plasma zone. The optics head as well as the probe rose a predetermined height after each printed layer under the assumption of a constant deposition thickness. However, since the over- and under-building are common occurrences in the DED process, the deposition layer may be slightly thinner or thicker than the preset layer thickness. In that case, the relative location between the probe and plasma will change. Then the probe would focus on a different zone of plasma which can cause a change of spectra intensity or even spectral profile^29^. Consequently, the change of spectra caused by the variability of deposition thickness induced errors for porosity recognition. Besides, the thickness deviation would increase the error of post-build porosity characterization. Thus, spectra data may be labeled incorrectly and this error accumulated with layers. These limitations are great challenges for online recognition of DED. However, this anomalous thickness variability can be eliminated using a height control^30^.

### Visualization of the important spectral feature

The relative importance of the eighteen spectral features during the classification process was recorded by the RF classifier. This information can describe how closely spectral features are associated with the printing quality and help to reveal the fundamental physics of defects recognition. Table 5 lists the four most important features reported by the RF classifier. It can be seen that the intensities of Al (396.054 nm) and Fe (414.234 nm) emissions, the kurtosis of spectra in two wavelength ranges (508–518 nm and 484–490 nm) are mostly associated with the porosity defects.

**Table 5:** The top four important features reported by the RF classifier.

N0	Feature	Importance value	Physical meaning
1	*I*_414.234_	0.102	The emission intensity of Fe I
2	*k*_(508–518)_	0.092	Kurtosis of spectra in the wavelength range 508–518 nm
3	*K*_(484–490)_	0.082	Kurtosis of spectra in the wavelength range 484–490 nm
4	*I*_396.054_	0.064	The emission intensity of Al I

**Figure 8:**
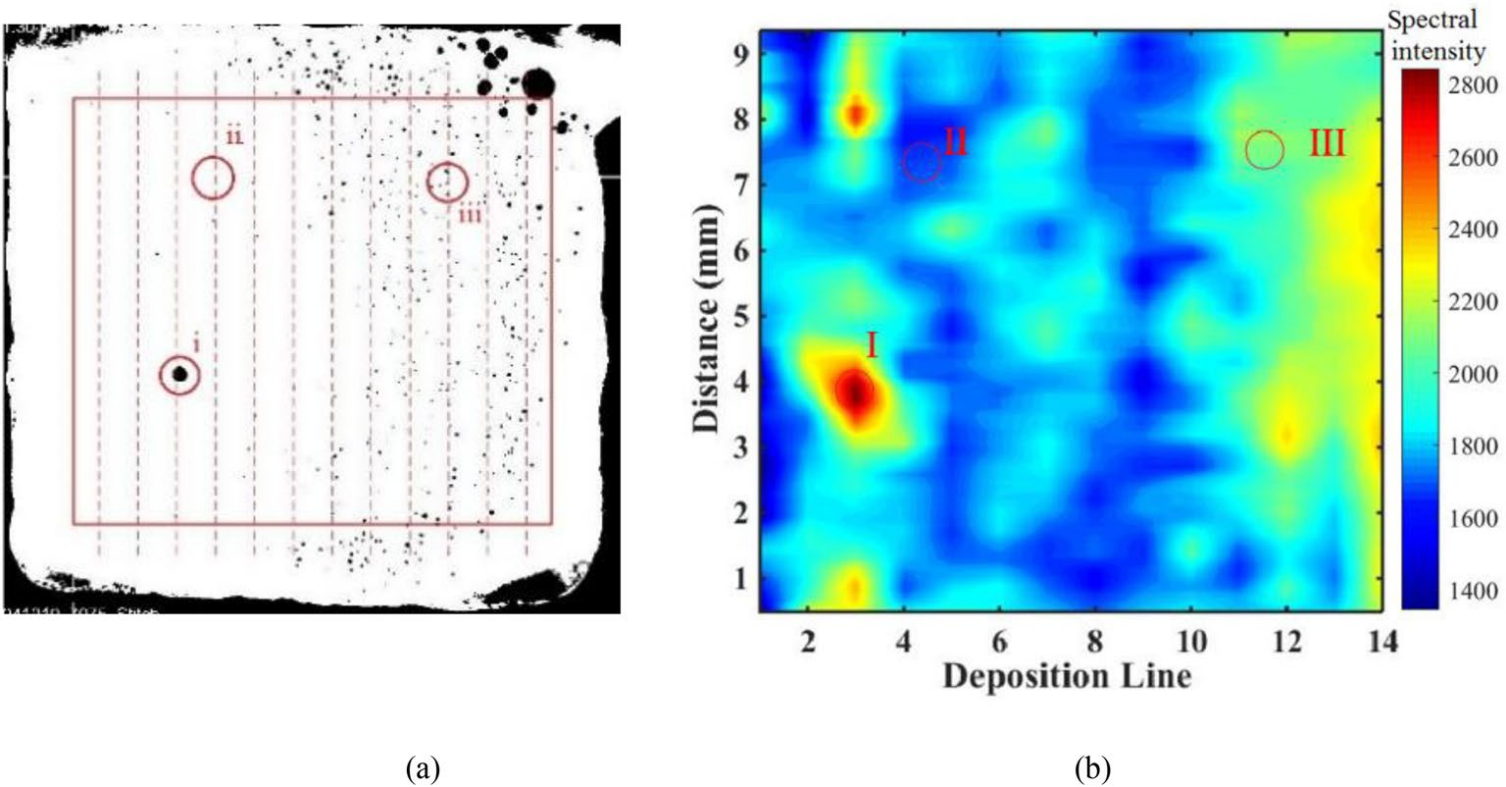
The comparison of overlaid binary CT image **(a)** with the spectral feature contour **(b) **of the second layer.

The distribution of the most important features (*I*_414.234_) for the second deposition layer was compared with the porosity distribution getting from the CT scans in this layer, as shown in Fig. 8. The *I*_414.234_ vector of this layer is reshaped to a matrix in sort of printing path in which each row of the matrix corresponding one printing line and mapped to a contour as shown in Fig. 8b. In the contour image the horizontal axis represents the printing line numbers, the vertical axis represents the distance from the lower deposition edge, and contour color represents the amplitudes of spectral feature *I*_414.234_. The binary CT images of this layer are overlaid together so that all pores produced in this deposition layer can be shown in one image as Fig. 8a. As can be seen, the second half of the deposition layer is more porous which corresponds to a higher *I*_414.234_. The first half part, by contrast, has a lower porosity rate which corresponds to lower *I*_414.234_. The spectral feature (*I*_414.234_) increased markedly when pores are produced, especially the large pores. Therefore, the selected important spectral features match well with the porosity rate of the deposition. The significant increase of Fe spectral intensity indicates that more Fe elements were evaporated and excited in the plasma when pores present.

## Discussion

**Figure 9:**
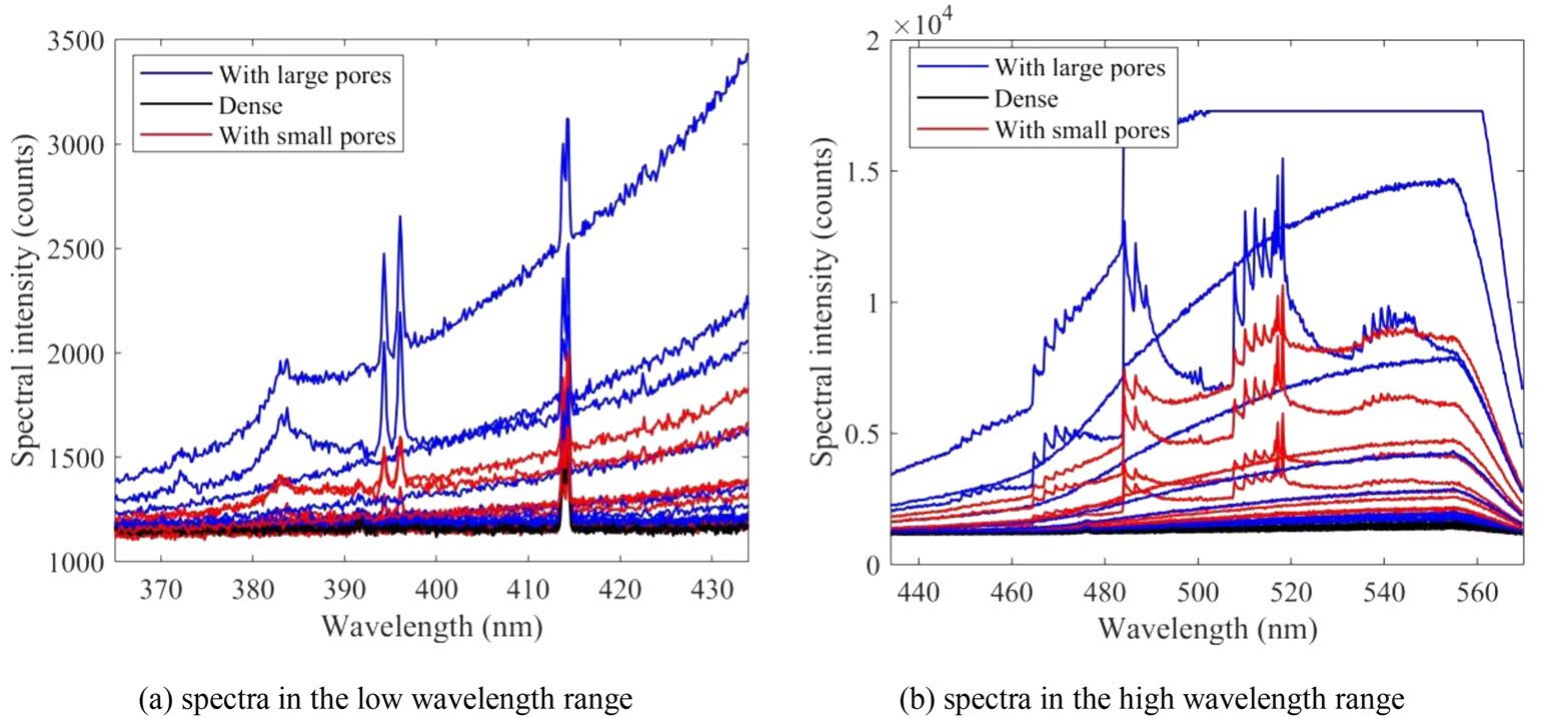
Comparison between spectra collecting from areas with a large random-pore (blue lines), without pores (black lines), and small concentrated pores (red lines).

Figure 9 presents the specific spectra taken from areas with large pores (blue lines), without pores (black lines), and small concentrated pores (red lines), respectively. As shown, emission lines as well as the background continuum of spectra for depositions with pores are much stronger than those for fully dense depositions. Intense metal emission lines are observed in the spectra when pores are produced, especially for the spectra corresponding to the large pores. The large pore was randomly produced around dense areas and is believed to be randomly caused by abnormal conditions, for instance, unstable laser input or metal spatter. These abnormal conditions create an active environment in which more elemental emissions are excited. As shown in Fig. 9, small pores are mostly concentrated in the second half part of the deposition layer. That is because the temperature of the build surface kept increasing during the printing process of the aluminium alloy, which increased the unstable depression within the molten pool and in turn entrapped gas as pores. Moreover, another factor for the pore formation is the metal evaporation under the high-temperature conditions. It has been proved that the evaporation of alloy elements in materials influences the convection dynamics as well as the uptake of hydrogen in the melted alloy which results in the formation of pores^28^. On the other hand, the high temperature and increased evaporation of alloy elements contribute to the strong intensity of spectra.

In summary, emission spectroscopy is an excellent in-situ monitoring technique for AM not only for academic research but also for commercial use. It is cheap and easy to access and can provide a large amount of fundamental information about the AM process. The multiple functions including composition recognition and defects detection make emission spectroscopy suitable for developing a cost-efficient monitoring system. Despite its obvious benefits, it is important to note the limitations of this technique. As can be shown in “Porosity recognition performance” section, spectra collection is highly sensitive to the relative position of the sensor, thus deposition thickness needs to be strictly controlled when using spectroscopy in the DED system. Furthermore, some kinds of material may not be easy to emit line emissions which result in a low signal-to-noise ratio of the spectra signals, especially when the system working under a low laser power.

## Conclusions

This work investigated the application of combined artificial intelligence and plasma emission spectroscopy for in-situ porosity recognition of additive manufacturing. Spectra signals synchronously collected during DED for 7075-Al alloy were analyzed and correlated with the quality of deposition which is characterized by the 3D X-ray CT scans. An RF classifier was developed and trained by examples consisting of spectral features and ground-truth deposition qualities with a resolution of 1.2 × 1.2 mm in the XY plane. This classifier achieved a porosity recognition precision up to 83% for the examples from the training layers or their adjacent layers. However, with the printing layer going up the recognition precision decreased to a low level which is mainly caused by the variability of deposition thickness.

The importance values of eighteen spectral features for porosity recognition were recorded by the RF classifier. The comparison of the most important spectral feature (*I*_414.234_) with the porosity distribution presents a good response to the random pores and concentrated small pores. Thermal accumulation and abnormal conditions increase the evaporation of element and the temperature of plasma which increase the porosity rate in the part as well as the intensity of spectra.

This work shows the potentials as well as the challenges of plasma emission spectroscopy applying on the in-situ quality monitoring of laser AM. It is significant for facilitating the online quality assurance and understanding the physical phenomena in the laser AM process. In future work, parts will need to be printed with strict deposition thickness control and used for further verification of the proposed method in this study. Deep learning classification models, such as long short-term memory models, may also be utilized to mine comprehensive and efficient information from spectra for defects recognition of AM.
